# Incidence, predictors, and timing of post-operative stroke following elective total hip arthroplasty and total knee arthroplasty

**DOI:** 10.1371/journal.pone.0239239

**Published:** 2020-09-17

**Authors:** Monique S. Haynes, Kareme D. Alder, Kirthi Bellamkonda, Lovemore Kuzomunhu, Jonathan N. Grauer

**Affiliations:** 1 Department of Orthopaedics and Rehabilitation, Yale School of Medicine, New Haven, CT, United States of America; 2 Department of Surgery, Washington University, Seattle, Washington, United States of America; Cleveland Clinic, UNITED STATES

## Abstract

**Background:**

Postoperative stroke is a rare but potentially devastating complication following total hip arthroplasty (THA) and total knee arthroplasty (TKA). The purpose of the current study was to determine the incidence, independent risk factors, and timing of stroke following THA and TKA utilizing the National Surgical Quality Improvement (NSQIP) database.

**Methods:**

Patients who underwent elective primary THA and TKA were identified in the 2005–2016 NSQIP database. Thirty-day postoperative strokes were identified, timing was characterized, and an incidence curve was created. Multivariate analyses determined the independent predictors of these strokes.

**Results:**

Of 333,117 patients identified, 286 (0.09%) experienced a stroke. Given that THA vs TKA was not a univariate predictor of stroke, the two procedures were considered together. The majority (65%) of strokes occurred before discharge. Of the strokes observed, 25% occurred by postoperative day one, 50% by postoperative day two, and 75% by postoperative day nine. Independent risk factors for postoperative stroke were: age (60–69 years old odds ratio [OR] = 4.2; 70–79 years old OR = 8.1; ≤80 years old OR = 16.1), higher American Society of Anesthesiologists (ASA) score (ASA≥3 OR = 1.7), and smoking [OR = 1.6).

**Conclusion:**

The incidence of stroke after THA/TKA was low at 0.09%, with the majority occurring prior to discharge and half occurring by postoperative day two. Patients who were older, sicker, or who were smokers were at greater risk of postoperative stroke. These findings can be used to council patients and to optimize patient care.

**Level of evidence:**

Level III, Retrospective comparative study.

## Introduction

Postoperative stroke is a rare but potentially devastating complication following total hip arthroplasty (THA) and total knee arthroplasty (TKA). In the United States, stroke has been identified as a perioperative complication following these procedures with a prevalence of less than one percent [1]. However, given the rising number of lower extremity joint replacements being performed in the United States annually, factors related to postoperative stroke in these patient populations warrant consideration [2, 3].

A study by Mortazavi et al. evaluated 18,745 patients who underwent THA or TKA at the Rothman Institute [4]. They found the incidence of stroke among this population to be 0.2% (36 patients from their cohort) [4]. Risk factors of perioperative stroke were found to be advanced age, history of cerebrovascular disease, history of heart disease, general anesthesia, and arrhythmia. Although a large patient population was studied, the relatively low number of patients with postoperative stroke may have limited the power to assess variables associated with stroke occurrence.

A National Inpatient Sample database study conducted by Rasouli et al. evaluated 1,762,496 patients who underwent THA or TKA [5]. Based on inpatient administrative data, they found the incidence of perioperative stroke to be 0.14%. Predictors of stroke were pulmonary circulation disorders, diabetes, arrhythmia, peripheral vascular disease, renal disease, and revision surgeries. Although the study had a significant sample size, it was inherently limited by the administrative data available in the dataset.

Finally, a study conducted by Bohl et al. utilized the 2005–2013 NSQIP database to examine timing of stroke following THA and TKA [6]. Of 124,657 patients, 118 had a perioperative stroke at a median of postoperative day two. While this study looked at associations between patient characteristics and timing of stroke, it did not include data now available from more recent years, and it also did not differentiate between strokes that occurred prior to discharge and those that occurred after.

Highlighting the impact of perioperative strokes after joint arthroplasty, prior studies have found perioperative stroke to be associated with 9%-39% rates of mortality [4, 5]. Thus, to add to the above-noted prior literature, the current study was performed to determine the incidence, independent risk factors, and, importantly, timing of stroke following THA and TKA utilizing the National Surgical Quality Improvement (NSQIP) database.

## Materials & methods

### Database

NSQIP is a registry database that contains data on hundreds of variables for over 400 participating medical centers in the United States [7]. The variables included in the database include demographics, preoperative comorbidities and functionality, and 30-day morbidity, mortality, readmission and reoperation following surgery [8]. The number of orthopedic patients included in the database has been increasing due to the increase in number of participating hospitals [7]. Exemption was granted by the institutional review board for studies utilizing this database.

Current Procedural Terminology (CPT) codes 27130 and 27477 were used to identify adult patients who underwent THA and TKA, respectively, for NSQIP 2005–2016. Patients who underwent emergent surgery or who were admitted to the hospital prior to their scheduled surgery were excluded from the present study. Patients with missing data were excluded from multivariate analyses.

### Demographic/Operative/Postoperative variables

Demographics and patient characteristics including age, sex, height, weight, American Society of Anesthesiologists (ASA) classification, preoperative functional status, smoking status and comorbidities such as chronic obstructive pulmonary disease (COPD), congestive heart failure (CHF), dyspnea, hypertension, diabetes, and renal insufficiency were obtained from NSQIP and coded into stratifications. Body mass index (BMI) was calculated from height and weight (mass[kg]/height[m]^2^). Operative variables were THA vs TKA and operative time.

The NSQIP database tracks patients for thirty days after surgery, regardless of discharge status. The postoperative outcome of interest for the present study was the occurrence of stroke post-surgery. Stroke was defined according to the NSQIP data user guide as an embolic, thrombotic or hemorrhagic vascular incident or stroke with motor, sensory or cognitive dysfunction that persisted for at least 24 hours. The timing of stroke was also abstracted from the database.

### Statistical analysis

The incidence and timing of perioperative stroke after THA and TKA were characterized. Given that THA vs TKA was not a univariate predictor of stroke, the two procedures were considered together. All statistical analysis was done using Stata. Tables and plots were constructed using Microsoft Excel.

### Univariate analysis

Demographic, comorbidity, and operative data were tabulated. Univariate chi-square tests were done to compare those who had and had not experienced postoperative stroke. Operation time was compared between the two groups using student t test. With Bonferroni correction, the alpha value was set at 0.003.

Similarly, chi-square and student t tests were conducted to compare all of the aforementioned demographic and comorbidity variables between patients who experienced stroke pre-discharge vs post-discharge. With Bonferroni correction, the alpha value was set at 0.003.

### Multivariate analysis

Multivariate logistic regression was done to identify independent predictors of postoperative stroke. The outcome variable was stroke and the model was controlled for age, sex, BMI, ASA, functional status, smoking status, COPD, CHF, dyspnea, hypertension, diabetic status, renal sufficiency, procedure type and operation time. Statistical significance was indicated by a p-value less than 0.05. Odds ratios, 95% confidence intervals and p-values were determined.

A multivariate logistic regression was also performed to identify independent risk factors of experiencing a stroke post-discharge vs pre-discharge. The model was controlled for age, sex, BMI, ASA, functional status, smoking status, COPD, CHF, dyspnea, hypertension, diabetic status, renal insufficiency, procedure type and operation time. Statistical significance was indicated by a p-value of 0.05. Odds ratios, 95% confidence intervals and p-values were determined.

## Results

### Demographics and comorbidities

In total, 333,117 patients met the inclusion criteria, 62% of which were TKA patients. Of this total population, 286 (0.09%) suffered a postoperative stroke. Demographics, comorbidities, and operative variables of those who suffered postoperative stroke and those who did not are shown in Table 1.

**Table 1:** Patient demographics and comorbidities.

Patient Demographics and Comorbidities			
	No Stroke	Stroke	
	332,831(100%)	286(100%)	Univariate Pvalue
**Age(mean±SD)**	**65.8±10.3**	**73.2±8.5**	<0.001
18-59	87,563(26.3)	16(5.6)	
60-69	122,000 (36.7)	76(26.6)	
70-79	92,070(27.7)	117(40.9)	
≥80	31,197(9.4)	77(27.0)	
**Sex**			0.738
Male	135,845(40.8)	114(39.9)	
Female	196,826(59.2)	172(60.1)	
**BMI(mean±SD)**	**32.0±7.0**	**31.5±6.4**	0.336
≤24 kg/m^2^	28,413(11.8)	32(15.8)	
25-29 kg/m^2^	68,288(28.5)	54(26.7)	
30-34 kg/m^2^	68,043(28.4)	58(28.7)	
≥35 kg/m^2^	75,172(31.3)	58(28.7)	
**ASA(mean±SD)**	**2.4±0.6**	**2.7±0.6**	<0.001
≤2	180,294(54.2)	89(31.2)	
≥3	152,210(45.8)	196(68.8)	
**Functional Status**			<0.001
Independent	327,475(98.5)	273(95.8)	
Dependent	4,981(1.5)	12(4.2)	
**Smoker**	34,379(10.3)	27(9.4)	0.622
**COPD**	12,379(3.7)	12(4.2)	0.670
**CHF**	942(0.3)	4(1.4)	<0.001
**Dyspnea**	18,166(5.5)	19(6.6)	0.378
**Hypertension**	205,542(61.8)	224(78.3)	<0.001
**Diabetic status**			<0.001
Non-insulin dependent	39,371(11.8)	46(16.0)	
Insulin dependent	12,456(3.7)	24(8.4)	
**Renal insufficiency**	640(0.2)	3(1.0)	0.001
**Procedure**			0.639
Total knee arthroplasty	206,971(62.2)	174(60.8)	
Total hip arthroplasty	125,860(37.8)	112(39.2)	
**Operation time**			
**Total knee arthroplasty(mean±SD)**	**93.0±38.0**	**93.8±37.3**	0.338
≤74 min	67,270(32.5)	56(32.2)	
75-99 min	69,813(33.7)	51(29.3)	
≥100 min	69,888(33.7)	67(38.5)	
**Total hip arthroplasty(mean±SD)**	**92.8±40.2**	**108.1±81.5**	0.266
≤74 min	44,026(35.0)	32(28.6)	
75-99 min	39,268(31.2)	35(31.2)	
≥100 min	42,566(33.8)	45(40.2)	

Shading represents significance at p<0.003

**Fig 1:**
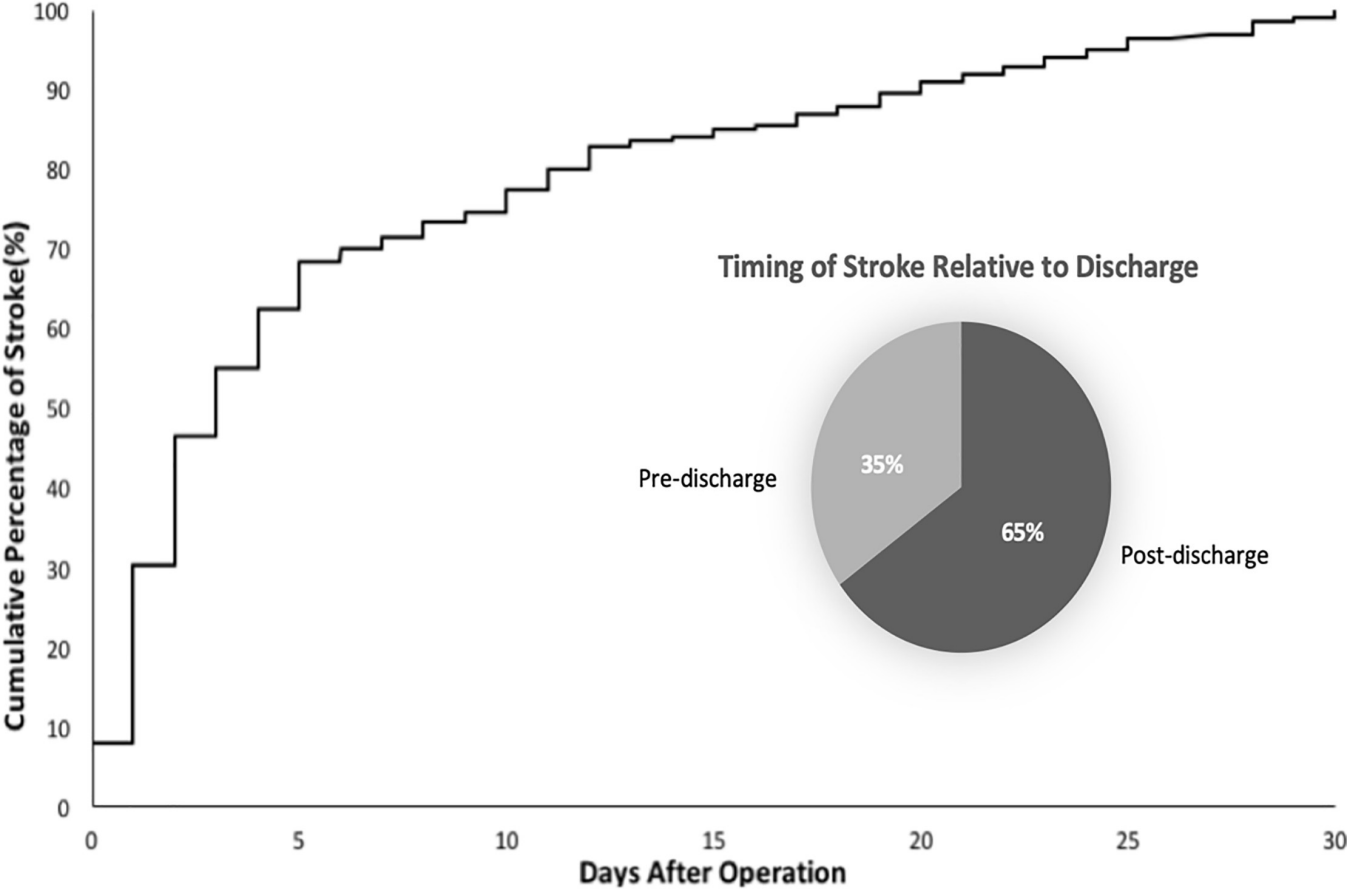
Cumulative percentage of stroke 30 days post-operation.

Of the strokes observed, 25% occurred by postoperative day one, 50% by postoperative day two, and 75% by postoperative day nine (Fig 1). The median day of stroke was postoperative day 3. Relative to discharge, 66% of strokes occurred before hospital discharge while 34% occurred after discharge. Demographics, comorbidities, and operative variables of those who suffered postoperative stroke pre- vs post-discharge are shown in Table 2.

**Table 2:** Patient demographics and comorbidities by stroke time.

Patient Demographics and Comorbidities by Stroke Time			
	Pre-discharge	Post-discharge	Univariate Pvalue
	Stroke incidence=0.09%		
	185(100%)	96(100%)	
**Age (mean±SD)**	**73.2±8.4**	**73.4±8.8**	0.750
18-59	10(5.4)	5(5.2)	
60-69	49(26.5)	27(28.1)	
70-79	79(42.7)	35(36.5)	
≥80	47(25.4)	29(30.2)	
**Sex**			0.881
Male	73(39.5)	37(39.2)	
Female	112(60.5)	59(61.5)	
**BMI (mean±SD)**	**31.8±6.2**	**29.7±7.1**	0.014
≤24 kg/m^2^	12(9.7)	20(26.7)	
25-29 kg/m^2^	34(27.4)	20(26.7)	
30-34 kg/m^2^	38(30.7)	18(24.0)	
≥35 kg/m^2^	40(32.3)	17(22.7)	
**ASA(mean±SD)**	**2.7±0.6**	**2.8±0.6**	0.556
≤2	60(32.6)	28(29.2)	
≥3	124(67.4)	68(70.8)	
**Functional status**			0.241
Independent	178(96.7)	90(93.8)	
Dependent	6(3.3)	6(6.2)	
**Smoking status**	17(9.2)	9(9.4)	0.959
**COPD**	7(3.8)	4(4.2)	0.875
**CHF**	3(1.6)	1(1.0)	0.697
**Dyspnea**	16(8.7)	2(2.1)	0.033
**Hypertension**	143(77.3)	77(80.2)	0.575
**Diabetic status**			0.302
Not diabetic	135(73.0)	78(81.3)	
Non-insulin dependent	32(17.3)	14(13.9)	
Insulin-dependent	18(9.7)	6(5.9)	
**Renal insufficiency**	2(1.1)	1(1.0)	0.976
**Procedure**			0.649
Total knee arthroplasty	115(62.2)	57(59.4)	
Total hip arthroplasty	70(37.8)	39(40.6)	
**Operation time**	** **		
**Total knee arthroplasty(mean±SD)**	**93.9±37.2**	**91.3±36.4**	0.490
≤74 min	36(31.3)	20(35.1)	
75-99 min	32(27.8)	19(33.3)	
≥100 min	47(40.9)	18(31.6	
**Total hip arthroplasty(mean±SD)**	**105.5±71.6**	**112.3±98.8**	0.305
≤74 min	23(32.9)	8(20.5)	
75-99 min	19(27.1)	15(38.5)	
≥100 min	28(40.0)	16(41.0)	

Shading represents significance at p<0.003

### Univariate analyses

Univariate chi-squared analysis showed that having a postoperative stroke was associated with older age (73.2±8.5 years vs. 65.8±10.3 years, P< 0.001), higher ASA score (2.7±0.6 vs 2.4±0.6, P <0.001), functional dependency (4.2% vs 1.5%, P < 0.001), CHF status (1.4% vs 0.3%, P<0.001), hypertension (78.3% vs 61.8%, P < 0.001), diabetic status (24.4% vs 15.5%, P<0.001), and renal insufficiency (1.0% vs 0.2%, P = 0.001) (Table 1).

A similar univariate analysis comparing patients who had stroke pre-discharge vs post- discharge showed no significant associations between timing of stroke and patient characteristics, comorbidities, or operative characteristics (Table 2).

### Multivariate analyses

**Fig 2:**
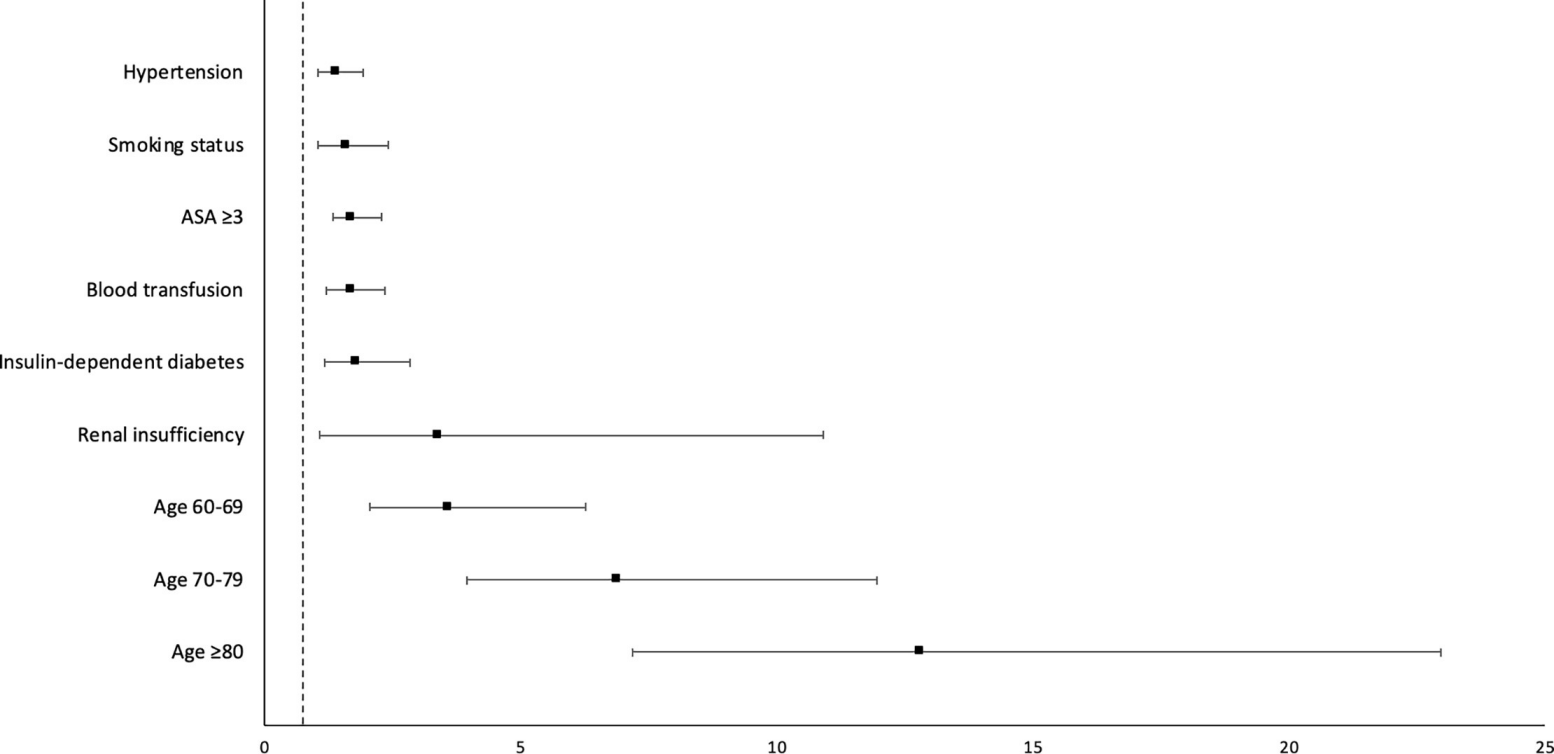
Multivariate analysis: Independent risk factors for perioperative stroke. *Crossing the vertical line at one indicates significance.

In the assessment of independent risk factors for developing postoperative stroke, multivariate analysis revealed: older age (Odds Ratio [OR] = 4.2 [95% CI = 2.13–8.36] for 60–69 years; OR = 8.1[95% CI = 4.10–15.97] for 70–79 years; and OR = 16.1 [95% CI = 7.89–32.88] for ≥80 years, P< .001), ASA score ≥3 (OR = 1.7 [95% CI = 1.23–2.30], P = .001), and smoking status (OR = 1.6 [95% CI = 1.06–2.42], P = .026). The occurrence of postoperative stroke was not dependent on sex, BMI, functional status, COPD, CHF, dyspnea, hypertension, diabetic status, renal insufficiency, or type of arthroplasty procedure (THA vs TKA) (P > .05 for each). Results of these analyses are shown in Table 3 and Fig 2.

**Table 3:** Multivariate analysis: Independent risk factors for perioperative stroke.

Multivariate Analysis: Independent Risk Factors for Perioperative Stroke			
Characteristic/Comorbidity	OR	95% CI	Multivariate Pvalue
**Age**			
60-69	4.2	2.13-8.36	<0.001
70-79	8.1	4.10-15.97	<0.001
≥80	16.1	7.89-32.88	<0.001
**Sex**			0.471
Female	0.9	0.68-1.20	
**BMI**			
25-29 kg/m^2^	0.7	0.47-1.15	0.183
30-34 kg/m^2^	0.9	0.56-1.37	0.558
≥35 kg/m^2^	0.9	0.57-1.47	0.721
**ASA**			0.001
≥3	1.7	1.23-2.30	
**Functional status**			
Dependent	1.8	0.93-3.62	0.079
**Smoking status**	1.6	1.06-2.42	0.026
**COPD**	0.5	0.22-1.18	0.116
**CHF**	1.9	0.47-7.80	0.370
**Dyspnea**	0.8	0.43-1.44	0.441
**Hypertension**	1.3	0.94-1.90	0.103
**Diabetic status**			
Non-insulin dependent	1.1	0.70-1.58	0.322
Insulin-dependent	1.6	0.95-2.84	0.075
**Renal insufficiency**	3.4	0.83-13.93	0.090
**Procedure**			
Total hip arthroplasty	1.1	0.84-1.50	0.444
**Operation time**			
75-99 min	1.1	0.74-1.48	0.804
≥100 min	1.3	0.90-1.77	0.804

Shading represents signfiicance at p<0.05

Controlled for age, sex BMI, ASA, functional status, smoking status, COPD, CHF, dyspnea, hypertension, diabetic status, renal insufficiency, procedure type and operation time

In the assessment of independent risk factors for developing postoperative post-discharge stroke, multivariate analysis revealed that BMI was the only significant independent predictor of timing of stroke (Table 4). Higher BMI was associated with a protective effect against having post-discharge stroke (OR = 0.4 [95% CI = 0.14–0.99], P = 0.047 for BMI 25-29kg/m^2^; OR = 0.3 [95% CI = 0.10–0.75], P = 0.011 for BMI 30–34 kg/m^2^; OR = 0.3 [95% CI = 0.08–0.75], P = 0.013 for BMI ≥35 kg/m^2^. The occurrence of post-discharge stroke was not dependent on age, sex, ASA, functional status, smoking status, COPD, CHF, dyspnea, hypertension, diabetic status, renal insufficiency, or type of arthroplasty procedure (THA vs TKA) (P > .05 for each). Results of these analyses are shown in Table 4.

**Table 4:** Multivariate analysis: Independent risk factors for post-discharge stroke.

Multivariate Analysis: Independent Risk Factors for Post-discharge Stroke			
Characteristic/Comorbidity	OR	95% CI	Multivariate Pvalue
**Age**			
60-69	0.8	0.18-3.66	0.797
70-79	0.7	0.16-3.30	0.689
≥80	0.7	0.15-3.40	0.677
**Sex**			
Female	1.1	0.59-2.17	0.705
**BMI**			
25-29 kg/m^2^	0.4	0.14-0.99	0.047
30-34 kg/m^2^	0.3	0.10-0.75	0.011
≥35 kg/m^2^	0.3	0.08-0.75	0.013
**ASA**			
≥3	1.4	0.72-2.88	0.300
**Functional status**			
Dependent	1.2	0.24-5.76	0.839
**Smoking status**	1.4	0.48-4.1	0.540
**COPD**	3.5	0.41-30.30	0.251
**CHF**	1.6	0.07-39.24	0.760
**Dyspnea**	0.2	0.02-1.41	0.099
**Hypertension**	1.0	0.45-2.06	0.914
**Diabetic status**			
Non-insulin dependent	0.4	0.15-1.23	0.115
Insulin-dependent	0.8	0.21-3.15	0.761
**Renal insufficiency**	0.9	0.04-21.30	0.970
**Procedure**			
Total hip arthroplasty	0.8	0.41-1.65	0.583
**Operation time**			
75-99 min	1.3	0.56-2.79	0.584
≥100 min	1.4	0.65-3.20	0.367

Shading represents significance at p<0.05

Controlled for age, sex BMI, ASA, functional status, smoking status, COPD, CHF, dyspnea, hypertension, diabetic status, renal insufficiency, procedure type and operation time

### Missing data

There was missing data upon analysis. Approximately 28% of the patient sample was missing data on age, sex, height, weight ASA score or functional status. For this reason, numbers listed in Table 1 may not add up to column totals due to missing data. Additionally, 5 of the 286 patients who had a stroke were missing data on either number of days from operation to discharge and /or number of days from operation to stroke. Therefore, multivariate analysis of risk factors for pre-discharge and post-discharge stroke was done using data from only 281 patients.

## Discussion

As the United States population ages, the demand for THA and TKA will continue to rise [9]. While clinical outcomes following THA and TKA are generally very good, post-operative complications must be taken into consideration; postoperative stroke is one such complication and has been associated with atrial fibrillation, myocardial infarction, and coagulopathy following surgery [10].

The current study found the risk of stroke after THA/TKA to be 0.09%. This was consistent with prior literature. Minhas et al. determined that 0.08% of patients undergoing elective TKA experienced a perioperative stroke, while Bateman et al. determined that 0.2% of patients who underwent elective THA suffered a perioperative stroke [11, 12].

The current study utilized multivariate analysis to determine factors associated with post THA/TKA stroke. These factors were: advanced age, increased ASA score, and smoking status. Advanced age is consistent with prior findings, including a study by Shobhit et al., which found that an age of 75 years or older was an independent risk factor for cerebral vascular accident(CVA) following elective orthopedic procedures [11]. Increased ASA also seems consistent with Shobhit el al’s findings that insulin-dependent diabetes, hypertension, history of transient ischemic attack, and COPD are risk factors for postoperative CVA, considering that ASA classification encompasses all of those factors. Smoking is consistent with this being a known risk factor for atherosclerotic vascular conditions.

Finally, the timing of post THA/TKA strokes was determined in the present study. Of the strokes observed, 25% occurred by postoperative day one, 50% by postoperative day two, and 75% by postoperative day nine. Although the majority of strokes (66%) occurred before discharge, 34% occurred post discharge. These finding are consistent with prior research suggesting that most postoperative strokes occur in the early postoperative setting but are notable for the fact that about one third of them occur post-discharge (when surveillance and assistance with care may be decreased). Prior research on postoperative stroke includes a 2017 NSQIP retrospective study that examined the timing of post total joint arthroplasty adverse events [6]. It found that the earliest occurring events were stroke and myocardial infarction, with median day of diagnosis for postoperative stroke being day 2 [6]. This median day finding was consistent with the results of a 2016 NSQIP study that examined risk factors for cerebral vascular accidents after elective orthopedic procedures [11]. Neither one of these studies, however, determined the timing of and risk factors for pre- vs post-discharge stroke.

In assessing independent risk factors for postoperative stroke, the present study found that only lower weight patients were at significantly greater risk than patients of higher BMI. While it seems counterintuitive that patients with increased BMI are less likely to suffer a stroke after discharge, this finding is supported by prior research. This research includes a study done by Thornqvist et al. in 2014, which examined the 30-day risks of cardiovascular adverse events and mortality associated with BMI following hip or knee replacement [13]. Using national Danish patient registries, the authors found that there was a U-shaped association between BMI and postoperative adverse outcomes, with underweight patients and obese patients having the worst outcomes [13]. The lowest risk for experiencing postoperative cardiovascular adverse events and mortality was among overweight patients [13].

By identifying patients at risk for stroke after THA/TKA, preventative strategies and monitoring can be focused on the most at-risk patients early in their postoperative course but should also be continued beyond discharge. This is important for optimizing care and minimizing associated healthcare cost burden [14, 15].

Strengths of the present study include characterization of timing of stroke using incidence curves and identification of predictors of pre- vs post-discharge stroke. An additional strength is utilization of the National Surgical Quality Improvement Program (NSQIP) database to follow patients 30-days after the index operation. Usage of NSQIP makes the conclusions of this study more generalizable, as NSQIP has been shown to be superior to other databases [7, 8, 16, 17].

The limitations of the present study, however, also stem from the utilization of NSQIP. NSQIP only affords postoperative data up to 30 days following the index operation [8]; thus, it is possible that some strokes related to these procedures may be missed if outside of this time window (even though the incidence was plateauing by the end of this observation period). Further, by using NSQIP, the study generated a demographic and comorbidity profile that was limited in scope by the variables currently available through the database.

## Conclusion

Overall, postoperative stroke was noted to occur in 0.09% of THA/TKA patients 30 days after surgery. The risk factors of postoperative stroke, predictors of timing of postoperative stroke, and the identification that 34% of these strokes occurred after discharge should be considered when optimizing patient care algorithms.
